# The Effect of Maternal Dietary Selenium Supplementation on Blood Antioxidant and Metabolic Status of Ewes and Their Lambs

**DOI:** 10.3390/antiox11091664

**Published:** 2022-08-26

**Authors:** Josip Novoselec, Željka Klir Šalavardić, Mislav Đidara, Maja Novoselec, Rosemary Vuković, Suzana Ćavar, Zvonko Antunović

**Affiliations:** 1 Faculty of Agrobiotechnical Sciences Osijek, University of J.J. Strossamyer in Osijek, Trg Sv. Trojstva 3, 31000 Osijek, Croatia; 2 Department of Biology, University of J.J. Strossmayer in Osijek, Cara Hadrijana 8, 31000 Osijek, Croatia; 3 Institute of Public Health Osijek-Baranja County, Franje Krežme 1, 31000 Osijek, Croatia

**Keywords:** ewes, selenium supplementation, antioxidant status, blood metabolites, transfer efficiency, lamb

## Abstract

This study investigated the effect of dietary selenium supplementation (organic and inorganic) of late-gestation ewes on blood selenium concentrations and metabolic and antioxidant status indicators in ewes and their lambs. In addition, the efficacy of selenium transfer from ewes to lambs during the suckling period was determined. The study was conducted on 30 Merinolandschaf ewes and their lambs and lasted four months. The feed mixture of the control group (group I) contained no added selenium, while the feed mixture of group II was enriched with 0.3 mg/kg of organic selenium sources and the third group with 0.3 mg/kg of inorganic selenium sources. In ewes and their lambs, selenium supplementation significantly (*p* < 0.01; *p* < 0.05) increased selenium concentration, glutathione peroxidase, and superoxide dismutase activity and decreased malondialdehyde concentration compared to the control group. Selenium supplementation had a positive effect on metabolism and hematological indicators in lambs. A positive correlation was found between antioxidant indicators in the whole blood of ewes and lambs. The good transfer of selenium from ewes to lambs was complemented by higher correlation coefficients when the feed mixture was supplemented with organic compared to inorganic selenium.

## 1. Introduction

Selenium is one of the most important trace elements, and its concentration must be adequate in animal feed. Metabolic disorders caused by selenium deficiency lead to lower productivity, health problems, and even the death of animals, and—as a consequence—financial problems for producers. A clear and specific clinical manifestation of selenium deficiency in ewes is muscular dystrophy, which primarily occurs in young lambs whose mothers were exposed to a lack of selenium [1]. Selenium deficiency leads to reduction of fertility, abortions, retained placenta [2], health problems in young animals, such as increased neonatal mortality, lower neonatal vitality, reduction of suckling reflex, and frequent occurrence of lamb diseases in general, and a weaker immune response of the organism [3,4]. Current regulations allow the amount of 0.3 mg of Se/kg of ewe diet in organic or inorganic form [5]. There are various methods of overcoming selenium deficiency [6], but sources of selenium supplements in feed can be classified into two categories: organic and inorganic. According to studies [7], the actual digestibility of selenium from feed containing selenite (inorganic form) in ewes is about 50%, while the digestibility of selenium yeast (organic form) would be about 66%, considering the difference in metabolism. Selenium efficiently crosses the placental barrier into fetal tissue and enters colostrum and milk, so provision of Se to the dam during gestation and early lactation is an effective method to meet Se requirements for newborn lambs [8,9]. Selenium concentration in soil, and thus in plants, is low in many parts of the world, including the Pannonian Basin region, to which Croatia belongs [10]. Consequently, selenium deficiency is found in goats and kids in Croatia [11]. The most studied selenoprotein belongs to a group of glutathione peroxidase (GSH-Px) [12], which plays a key role in the antioxidant system. Correlation between GSH-Px activity in tissue and selenium intake has been established [13]. Selenium supplementation of livestock diets has the potential to improve the oxidative stability of meat and subsequently nutritive value and flavor of meat products. Glutathione peroxidase catalyzes the reduction of lipid and hydrogen peroxidases to less harmful hydroxides by the oxidation and subsequent reduction of selenocysteine (SeCys), which is the active center of this enzyme. Oxidative stress and the enzyme superoxide dismutase (SOD) have a significant role in the pathogenesis of cardiovascular and infectious diseases, cancer, diabetes, neurodegenerative disorders, fibrosis, hemolysis, and aging hydrogen peroxide, and are an important factor in the antioxidant defense [14]. Results of many studies [15,16,17,18] indicate that Se supplementation may affect SOD activity in animals. Biochemical and hematological indices give us a comprehensive insight into the nutritional and health status of animals, especially during the demanding period after lambing and suckling. Therefore, this research aimed to evaluate and compare the selenium-transfer efficiency from mother ewes to lambs considering the source (form) of supplement in feed, selenized yeast, or sodium selenite, and to determine the level of oxidative stability and general health of the lambs.

## 2. Materials and Methods

### 2.1. Design of Experiment and Treatments

This research was conducted on 30 gestational Merinolandschaf breed ewes at the commercial farm Ursić in Zlatna Greda (Osijek Baranja County, Croatia).

The study was performed on 30 ewes of the Merinoland breed. The ewes were in their fourth lactation, healthy, and in good physical condition. The research started in autumn by selecting the late-gestation ewes (90th day of gestation) with a single lamb, and continued during the spring of next year with the same ewes after lambing, including their lambs during the suckling period. Gestation was detected with a Tringa 50S ultrasound machine (PIE Medical, Maastricht, Netherlands). In total, the study lasted four months: two months with ewes in the late-gestation period and two months with the same lactating ewes and their lambs during the suckling period. Ewes were divided into three groups of 10 highly gestational animals each (last trimester of gestation). After lambing, the lambs remained with their mothers until the end of the research and were allowed to suckle ad libitum. Each group consisted of 10 healthy ewes and 10 lambs in total. The control group of animals (group I) was fed a meal that consisted of 300 g/day/animal feed mixtures without selenium supplement and 150 g/day/animal of barley (132 g/kg dry matter). The second group of animals (group II) was fed a meal consisting of 300 g/day/animal of feed mixtures additionally fortified with 0.3 mg/kg organic selenium (Selplex^®^, Alltech, Zagreb, Croatia) and 150 g/day/animal of barley (132 g/kg dry matter). The third group of animals (group III) was fed a meal consisting of 300 g/day/animal of feed mixtures additionally fortified with 0.3 mg/kg inorganic selenium (sodium selenite) and 150 g/day/animal of barley (132 g/kg dry matter). All ewes, and later lambs, were fed feed mixture and barley individually in separated feeding troughs, offered in two portions, during morning and evening feeding. No refusals of concentrate and barley were observed. All three groups of animals were fed with Alfalfa hay ad libitum (~1.20 kg/animal/day; 1032 g/kg dry matter). Animals had access to fresh water ad libitum. The ingredients and chemical composition of the basal feed mixture are shown in Table 1.

### 2.2. Chemical Composition of Feed

Four replicates of 10 g of concentrate mixture, barley, and hay were sampled, dried at 60 °C, ground to 1 mm particle size using a cutting mill (Microtron MB 550; Kinematica, Luzern, Switzerland), and analyzed for chemical composition (Table 1). Standard methods [19] were used to determine the basic chemical composition of feeds. Crude protein concentration was estimated from nitrogen content according to the Kjeldahl method [20]. Crude lipid concentrations were analyzed according to [21] using the Extraction System B-811 (Buchy, Flawil, Switzerland). Crude fiber concentrations were determined using the Weende method. Finally, crude ash concentrations were determined by incinerating the feed samples at 550 °C for 6 h. Samples of the feed mixture for the determination of selenium content were dried and ground to a fine powder using a heavy metal-free ultracentrifugal mill (Retsch RM 200, Haan, Germany) or a knife (GM 200, Haan, Germany). Samples were digested with 10 mL of a 5:1 HNO_3_ and H_2_O_2_ mixture at 180 °C for 60 min in a microwave oven (CEM Mars 6, Matthews, NC, USA). The concentration of selenium in solutions of digested samples was determined by inductively coupled plasma (ICP, PerkinElmer Optima 2100 DV, Waltham, MA, USA). Each batch of samples run on the ICP was analyzed with internal pooled plasma control and with the reference material prepared in the same way as for the other samples. In the feed mixtures of ewes and lambs, the following amounts of selenium per dry matter (DM) were determined by laboratory analysis: 0.092 ± 0.01 mg/kg in the control group, 0.30 ± 0.05 mg/kg in the second and 0.299 ± 0.07 mg/kg in the third group. Milk samples were taken at 7.00 a.m. before feeding on the 23rd day of lactation. Before collecting raw milk, sampling bottles were soaked in 20% HNO_3_ for 24 h and washed with deionized water to avoid possible contamination. The digestion of milk samples was carried out according to the method described by [22]. Each milk sample (3 mL) was transferred to a 60 mL Teflon digestion vessel and then optimized volumes of 6 mL of 70% nitric acid and 1 mL of 30% hydrogen peroxide were added, and the mixture was carefully shaken and held for 10 min before the vessel was closed. Samples were subjected to closed microwave digestion using the optimized microwave digestion program in the following order: 50 W, 165 °C (10 min); 80 W, 190 °C (20 min); and 0 W, 50 °C (10 min), performed on the Mars 6 microwave system (CEM, Matthews, NC, USA). After heating, the samples were cooled to room temperature and the digestion vessels were carefully opened in a digester. The digest was diluted to 25 mL with deionized water and subjected to a pre-reduction step before analysis as per [23]. A portion (20 mL) of the sample was placed in a purified 125 mL beaker and 20 mL of the concentrate HCL was slowly added. The solution was then transferred to a 50 mL polypropylene autosampler tube, which was diluted to the 50 mL mark with deionized water. The samples were ready to run with inductively coupled plasma (Optima 21000 DV, PerkinElmer, Waltham, MA, USA).

### 2.3. Body Weight of Lambs

Body weight of lambs was measured at birth and 23 and 63 days by the Kern EOS 150K50XL animal platform scale (Kern & Sohn, Balingen, Germany).

### 2.4. Collection and Analysis of Blood Samples

Blood samples were collected from late-gestation and lactating ewes (on days 14 and 23 relative to lambing) and from their lambs during suckling at an average age of 23 and 63 days. Venoject sterile vacuum tubes (Leuven, Belgium) were used for blood collection from the jugular vein. After blood sampling, lamb serum was separated by centrifugation (10 min) at 1609.92× *g* and analyzed in an Olympus AU640 analyzer (Olympus, Tokyo, Japan). Mineral concentrations (iron—Fe; inorganic phosphorus—P; calcium—Ca; sodium—Na; potassium—K; chloride—Cl), biochemical parameters (glucose, urea, total proteins, cholesterol, albumin, globulin, triglyceride, high-density lipoprotein (HDL), low-density lipoprotein (LDL), glutathione peroxidase (GPX), and superoxide dismutase (SOD)), and hepatic enzyme activity (alanine aminotransferase—ALT; aspartate aminotransferase—AST; gamma-glutamyltransferase—GGT; creatine kinase—CK) were all determined in serum using Olympus System Reagents (OSR), manufactured and distributed by Olympus Diagnostica GmbH (Irish branch), (Lismeehan, Ireland). Content of globulin was determined as the subtraction of total protein and albumin. Hematological indicators (white blood cells—WBC, red blood cells—RBC, hemoglobin content—HGB, mean corpuscular volume—MCV, mean corpuscular hemoglobin—MCH, mean corpuscular hemoglobin concentration, and platelet count—PLT) were determined on the hematology 3 diff analyzer Sysmex pocH 100 iV (Sysmex Europe GmbH, Hamburg, Germany). The relative proportion of individual leukocyte types was determined on blood smears stained by the Pappenheim method after fixation in air. Differentiation of the white blood count was done using an Olympus BX 51^®^ microscope (Tokyo, Japan). In the whole blood of ewes and lambs, selenium, GSH-Px, SOD, and malondialdehyde (MDA) were determined. Electrothermal atomic absorption spectrometry with palladium as matrix modifier and Zeeman background (AAnalyst 600, PerkinElmer Instruments, Waltham, MA, USA) was used to determine selenium concentration in digested samples. Selenium concentration in ewes’ milk was determined in the digested samples by graphite atomic absorption spectrophotometry using palladium nitrate and magnesium nitrate as matrix modifiers and Zeeman background correction (AAnalyst 800, PerkinElmer Instruments, Waltham, MA, USA). The GSH-Px activity was determined using a commercial kit (RANSEL, RANDOX, London, UK) based on the method of [24]. GSH-Px activity in the blood of ewes and lambs was expressed as U/mL of whole blood and converted into μkat/L. Superoxide dismutase (SOD) was determined spectrophotometrically (505 nm) using the commercial kit RANSOD (RANDOX, San Diego, CA, USA) by measuring the decrease in absorbance during 3 min using a Lambda 2 UV-vis spectrophotometer (PerkinElmer, Rodgau, Germany). SOD activity in the blood of ewes and lambs was expressed as U/mL of whole blood. Oxidative stress was fortified by determining the concentration of lipid peroxidation products (LPO) in whole blood by measuring thiobarbituric acid reactive substances (TBARS), according to the method described by [25]. The method is based on the measurement of red pigment formed by the reaction of MDA, which is one of the products of lipid peroxidation and thiobarbituric acid (TBA).

### 2.5. Statistical Analysis

Mean values of the obtained research result indicators of ewes’ and lambs’ antioxidant status, as well as biochemical and hematological parameters of lambs, were calculated by the MEANS procedure in the computer program TIBCO Statistica^®^ 14.0.0. Data were analyzed by means of ANOVA, using feeding treatment as a fixed effect. Mean values were compared using Tukey’s test and differences between the groups were declared significant at *p* < 0.05. Effects of treatment (group I without selenium; group II 0.3 mg/kg of organic selenium feed supplement, group III 0.3 mg/kg of inorganic selenium feed supplement), times (late-gestation lactating ewes; lambs aged 23–63 days), repetitions and their interaction in the experimental period on the abovementioned indicators were analyzed using GLM repeated-measures ANOVA. Where significant differences were determined, the LSD post hoc test was performed. After that, correlation and regression equations were calculated.

## 3. Results

### 3.1. Effects of Selenium Supplementation on the Antioxidant Status Indicators in the Blood of Ewes

Adding selenium to late-gestation and lactating ewes’ feed mixture significantly influenced almost all indicators of antioxidant status. Significantly higher selenium concentrations were found in the whole blood of late-gestation and lactating ewes in group II (Selplex®, Zagreb, Croatia) and group III (sodium selenite) than in group I (without Se) (96.86:157.56:141.01 µg/L). The highest selenium concentration in blood of late-gestation ewes was determined in the group fed with the addition of organic selenium supplement, and a significant difference (*p* < 0.01) was found between group II and group III (Table 2). In lactating ewes, the highest concentration of selenium was found in group II (143.18 µg/L) and significantly (*p* < 0.05; *p* < 0.01) different from groups III and I (131.37:93.37 µg/L). The increase in the concentration of selenium in the whole blood of ewes, depending on its source (form) in feed, was accompanied by an increase in the activities of GSH-Px enzyme (Table 2). In groups II and III, significantly higher (*p* < 0.01) activity of GSH-Px was detected in the whole blood of late-gestation ewes (899.85:771.67 μkat/L) than the control group I (581.18 μkat/L). This tendency of growing concentration with significant differences (*p* < 0.01) was also observed in lactating ewes (569.08:1054.91:884.65 μkat/L) in groups I, II, and III. The addition of organic, compared to the inorganic, form of selenium in lactating ewes significantly (*p* < 0.01) increased the activity of GSH-Px in whole blood, while in the late-gestation ewes there were no significant differences (*p* > 0.05). As for the concentration of MDA in whole blood, its reduction was noticed as the concentration of selenium in the blood increased. In the blood of late-gestation ewes, the organic form of selenium influenced a significant decrease (*p* < 0.05) in MDA concentrations compared to the control group (20.13:32.49 nmol/mL) (Table 2). Statistically significant differences were not determined for the activity of SOD.

### 3.2. Effects of Selenium Supplementation on the Antioxidant Status Indicators in the Blood of Lambs

In brief, the body weight (birth weights:end of research) of lambs depending on dietary treatments was not significantly different (group I: 4.76 and 21.59 kg; group II: 4.92 and 21.08 kg; group III: 5.02 and 21.76 kg). Daily gains of lambs were 267.28, 256.35, and 265.69 g in the first, second, and third group. The highest, and significantly higher (*p* < 0.01), concentrations of selenium levels in the first (63.62:48.21 μg/L) and second sampling (89.23:58.62 μg/L) were found in lambs who, as well as their mothers, had the addition of organic selenium in feed mixture compared to the control group without selenium supplement (Table 3). Adding inorganic selenium to the feed mixture also affected the increase in selenium concentration (*p* < 0.05) in the blood of lambs compared to the control group without the addition of selenium in the feed mixture (56.76:48.21; 80.71:58.62 μg/L), and significant differences (*p* < 0.05) were found between organic and inorganic selenium supplement (Table 3). Significantly higher activity (*p* < 0.01) of GSH-Px was found in the blood of younger (1023.78:928.94:590.65 μkat/L) and older lambs (1017.51:857.83:596.80 μkat/L), whose mothers, just like them, had the addition of organic and inorganic selenium in their feed mixture compared with control. Significant differences (*p* < 0.05) related to the activities of GSH-Px in the blood of lambs were observed between organic and inorganic selenium supplement in the feed mixture. Significantly higher (*p* < 0.01, *p* < 0.05) SOD activity than the control group was found in the blood of younger (1183.13:1115.97:950.15 U/mL) as well as older lambs (1208:1223.01:1081.34 U/mL) with the addition of organic and inorganic selenium in feed mixture compared to the control group (Table 3).

### 3.3. Effects of Selenium Supplementation on the Blood Biochemical Indicators of Lambs

Most of the determined biochemical indicators were within the reference values [26], which we can associate with appropriate nutrition. The addition of selenium supplement (organic or inorganic) to the feed mixture for ewes and lambs had no significant effect on the mineral concentration in the blood of the lambs (Table 4). Significantly (*p* < 0.05) higher albumin concentration was found in younger lambs with the addition of organic and inorganic selenium in the feed mixture than lambs in the control group (28.86:30.18:30.17 gL^−1^). The globulin concentration (*p* < 0.05) in the blood of lambs aged 23 days supplemented with inorganic selenium in the feed mixture was significantly increased (22.80:26.44 gL^−1^) compared with the control group (Table 4). Significantly (*p* < 0.05) lower concentration of cholesterol was determined in lambs aged 23 days supplemented with inorganic selenium supplement compared to the control group with no selenium added (2.69:1.96 mmol L^−1^). Inorganic selenium supplementation caused a significant (*p* < 0.05) decrease in LDL concentration in lambs of both age-groups compared with the control group (0.96:0.54; 0.61:0.42 mmol L^−1^). Significantly decreased (*p* < 0.01) LDL concentration was determined in 63-day-old lambs’ blood supplemented with organic selenium (0.61:0.40 mmol L^−1^). The significant influence in selenium supplementation of the feed mixture on the activity of AST and GGT enzyme in the blood of lambs was determined. The organic and inorganic selenium supplement in the fodder mixture of ewes and lambs significantly (*p* < 0.05) affected the decrease in AST activity in the blood of lambs during the second sampling (185.20:148.38:121.34 UL^−1^). The lowest activity of the AST enzyme (in reference range) was determined in the third group with the addition of inorganic selenium. The GGT activity in the blood serum of lambs during the first sampling generally significantly (*p* < 0.01) decreased with the addition of selenium supplement (organic and inorganic) compared to the control group (128.18:86.34:84.60 UL^−1^).

### 3.4. Effects of Selenium Supplementation on the Blood Hematological Indicators of Lambs

Lambs’ hematological parameters in the blood are shown in Table 5. Blood hematological indicators of lambs were in the reference range according to [27].

Inorganic selenium significantly (*p* < 0.05) affected the increase in the number of leukocytes in lambs at the first sampling compared to lambs from the control group (8.04:9.91:13.07 × 10^9^/L), while at the second sampling there was an increasing trend, but without statistically significant differences. The average value of mean corpuscular volume (MCV) was the highest in lamb blood at the first sampling with the addition of organic selenium, and it was significantly (*p* < 0.05) higher than in lambs from the control group (39.23:41.94:39.46 fL). The highest value of mean corpuscular hemoglobin (MCH) in erythrocytes was determined in older lambs that received organic selenium supplementation in a feed mixture and was significantly (*p* < 0.01; *p* < 0.05) higher than in lambs supplemented with inorganic selenium, i.e., lambs of the control group (12.74:13.71:11.30 pg). The highest mean value of MCHC in erythrocytes was found in lambs supplemented with organic selenium and was significantly different (*p* < 0.01) from lambs supplemented with inorganic selenium (335.36:360.66:229.90 g/L). In general, selenium addition (organic and inorganic) in lamb feed mixture significantly (*p* < 0.01) decreased platelet count (1275.20:873.71:689.24).

The analysis of the relative share of individual morphological forms of leukocytes in the blood of the lambs, presented in Table 5, shows that significant (*p* < 0.05) changes occurred only in the relative share of eosinophilic granulocytes, which were significantly higher in the lambs fed organic selenium supplement.

### 3.5. Selenium Transfer from Ewes to Lambs

**Figure 1:**
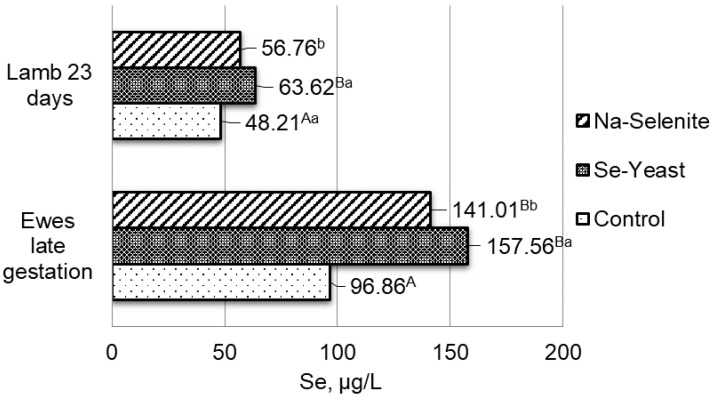
Concentration of selenium in whole blood of late-gestation ewes and 23-day-old lambs. I-control group; II-addition of organic selenium supplement; III-addition of inorganic selenium supplement; Se-selenium; ^a, b,^ *p* < 0.05; ^A, B,^ *p* < 0.01 differences between treatments.

**Figure 2:**
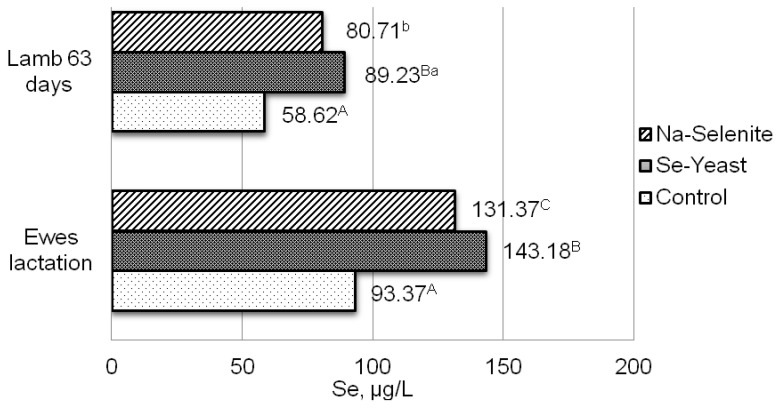
Concentration of selenium in whole blood of lactating ewes and 63-day-old lambs. I-control group; II-addition of organic selenium supplement; III-addition of inorganic selenium supplement; Se, selenium; ^a, b,^ *p* < 0.05; ^A, B, C,^ *p* < 0.01 differences between treatments.

A significant increase in the selenium concentration in ewes’ milk that had selenium supplement in the feed mixture was determined: 15.85 ± 1.62; 28.73 ± 1.35, and 20.82 ± 1.90 mg L^−1^ in the first (control), the second (organic selenium), and the third (inorganic selenium) groups of ewes. The increased selenium concentration in the blood of ewes was followed by an increase in selenium concentration in the blood of lambs (Figure 1 and Figure 2). Selenium supplement in the feed mixture of late-gestation ewes increased the concentration of selenium by 62.66% when the source was organic (group II), i.e., 45.58% when the source was inorganic (group III) compared to the control group (group I) without selenium supplement (Figure 1). After the first sampling of 23-day-old lambs, an increase of 31.96% in the concentration of selenium in the blood with the addition of organic (group II) or 17.73% with the addition of inorganic (group III) sources of selenium in the feed mixture (Figure 1), was also determined. In lactating ewes, the addition of organic selenium in feed mixture (group II) increased the concentration of selenium in whole blood by 52.21%, and the addition of sodium selenite (group III) by 40.30% compared to the control group (group I) without selenium supplement (Figure 2). As expected, the concentration of selenium in the whole blood of lambs after the second sampling (aged 63 days) also increased by 52.21% with the addition of organic and by 37.68% with the addition of inorganic selenium supplement in the feed mixture (Figure 2) compared to the control group. It may be noted that the concentration of selenium in the blood of lambs after the second sampling, compared to the first, clearly and better followed the level of selenium in the blood of ewes. These results suggest that the level of selenium in lambs highly depends on the level of selenium in the milk of mothers.

### 3.6. Correlation and Regression Analysis between the Content of Antioxidant Parameters in the Blood of Ewes and Their Lambs

According to the analysis of the correlation and regression line, there was good connectivity between antioxidant parameters in the whole blood of ewes, i.e., their lambs, which is indicated by the positive correlation coefficients (Table 6, Table 7 and Table 8).

The organic selenium supplement significantly increased the activity of GSH-Px and SOD (*p* < 0.01) and decreased MDA in the whole blood of ewes and their lambs in both samplings. High correlation coefficients point to the quality selenium transfer from ewes to lambs and its impact on antioxidant indicators. Those correlation coefficients were significant for the activity of GSH-Px and MDA concentrations in whole blood of ewes and lambs from group II in the first sampling (r = 0.91, r = 0.98; Table 6 and Table 8).

## 4. Discussion

### 4.1. Effects of Selenium on the Antioxidant Indicators in Ewes as Dams

According to [28], the optimal concentration of Se in the blood of ewes is 120–150 µg/L, while values from 25 to 50 µg/L are considered deficient [29]. In the present research, the concentration of Se in the blood of late gestation and lactating ewes was optimal in group II and group III (157.56; 141.01 and 143.18; 131.37 μg/L). Blood Se concentrations of late-gestation and lactating ewes determined in group I of the present study indicate that ewes reared in this area have physiologically lower insufficient Se concentrations. Panev et al. [30], in compliance with our results, found a significant (*p* < 0.01) increase in the concentration of selenium and GSH-Px activity in the blood of ewes after adding selenium supplements to feed. Contrary to our results, the authors did not find significant differences in the concentrations of selenium and GSH-Px activity in the blood of ewes with respect to organic or inorganic selenium supplement in the feed. Misurova et al. [31] determined a similar increase in the concentration of selenium and GSH-Px activity in the blood of pregnant goats. The above authors found significantly higher (*p* < 0.05) concentrations of selenium (177.2; 159.0; 111.4 μkat/L) and activity of GSH-Px (1154.6; 1011.6; 581.9 μkat/L) in the blood of goats fed with rations supplemented with sodium selenite or selenium lactate complex than in the control group. Hall et al. [32] found an increase in selenium concentration in the whole blood of ewes fed 4.9 mg Se per week in the diet, but the increase depended on the selenium source (form) and the production stage of the animals, as in the present study. Steen et al. [33] conducted similar research on ewes fed rations in which 20 mg per kg of an organic or inorganic form of selenium was added. Two months after the start of the experiment, both groups of ewes had, in general, a higher concentration of selenium in whole blood than the period prior, without the addition of selenium. In compliance with our results, the ewes that were fed with the organic selenium supplement had a significantly higher concentration of selenium in whole blood (0.28 mg/g) than those that were fed with the inorganic selenium supplement (0.24 mg/g). However, [34] noticed an increase in the concentration of selenium in the blood of ewes only seven days after starting the treatment with sodium selenide. The concentration of selenium in the blood of ewes prior to the treatment was 0.038 mg/mL, which is below the reference values, which, according to [35,36] range from 0.071 to 0.426 mg/mL. After sodium selenide treatment, the selenium concentration in the blood serum of ewes increased significantly (*p* < 0.05) and was within the reference values (0.175 mg/mL). Juniper et al. [37] found a significant selenium concentration increase (*p* < 0.001) in the whole blood of dairy cows, heifers, calves, and lambs after adding high doses of selenium yeast (6.25; 6.74; 5.84; 6.63 mg/kg dry matter) to their food compared to the basic food without added selenium. The mean value of selenium concentration in whole blood increased to a maximum value of 716, 1505, 1377, 724 ng Se/mL in dairy cows, heifers, calves, and lambs. The GSH-Px activity was higher (*p* < 0.05) in animals whose feed was supplemented with selenium yeast than in the control group, which did not have the addition of selenium, in agreement with present study. The MDA value in the study by [37] decreased when the blood selenium concentration increased, as in the present study. Significantly lower MDA was found in the blood of late pregnant ewes fed organic selenium rations compared to the control group (*p* < 0.05). A similar result was obtained for MDA levels in the blood of lactating ewes, but without significant difference (*p* > 0.05). Pavlata et al. [38] found a significant increase in selenium concentration (*p* < 0.01) in pregnant goats that received the addition of organic and inorganic Se in the feed mixture compared to the control group (without selenium addition). There were no significant differences between the experimental groups. GSH-Px activity in the blood of goats was also higher in all experimental groups than in the control group. The results of our study are in agreement with those of [39], who reported that the supplemental administration of Se in the diet of ewes after parturition for a period of four weeks increased the Se concentration in the milk of the ewes and in the tissue and blood of the lambs.

### 4.2. Effects of Selenium on the Antioxidant Indicators of Lambs

In agreement with the present study, the addition of Se to the maternal diet increased Se concentration in serum and colostrum of mares and goats [40,41]. Juniper et al. [13], in agreement with the present study, found a significantly higher selenium concentration (*p* < 0.001) in the whole blood of lambs after the addition of selenium yeast to their diet. Faixova et al. [42] found a significant (*p* < 0.001) increase in the selenium concentration and GSH-Px activity in the blood of lambs by adding 0.3 mg of selenium from selenium yeast to lambs’ feed. Antunović et al. [43] examined the effect of adding organic and inorganic selenium supplement to lambs’ feed on its concentration and GSH-Px activity in blood. Selenium concentration and GSH-Px activity in blood were significantly higher (*p* < 0.01) in lambs with the addition of organic and inorganic selenium than in the control group. The authors also found higher selenium concentrations (*p* < 0.01) and GSH-Px activity (*p* < 0.05) in the blood of lambs with the addition of organic than the inorganic form of selenium in the feed. Similar results were found in other studies [33,44]. Shi et al. [45] found significantly higher selenium concentration (*p* < 0.05) in male kids fed diets with selenium supplementation (sodium selenite and selenized yeast) after 90 days of the experiment than the control group of kids. GSH-Px and SOD activity was significantly higher (*p* < 0.05) in both groups of kids fed the selenium addition than the control. The MDA significantly decreased (*p* < 0.05) in both groups of kids fed with selenium addition compared to the control. In agreement with the results of the present study in [46], feeding 1.2 mg Se to growing lambs significantly increased GSH-Px and reduced serum MDA concentration. Vignola et al. [47] found no significant effect of selenium addition in the diet of lambs on lipid peroxidation or MDA content. However, by selenium supplementation, a declining trend of MDA in muscles and fat was noticed. Padmaja et al. [48] reported that selenomethionine is an effective consumer of oxidation peroxynitrates, which are the product of nitrate oxide and superoxide and are capable of oxidizing a wide range of biomolecules, including lipids, proteins, and nucleic acids. Although with a shorter pause, the enzyme activity corresponds to the addition of selenium to ewes’ food, it also depends on the previous deficiency and the time required for protein synthesis [49]. These results suggest that there is a positive connection between the GSH-Px activities and the concentration of selenium in blood of lambs whose mothers had the addition of selenium in food, which was previously found by [50]. Juniper et al. [13] found higher GSH-Px activity (*p* = 0.0114) in lambs fed a selenium supplement in the diet compared to the control group without the supplement. However, they did not determine the effects of selenium origin (organic or inorganic) on GSH-Px. Although previous research established that the concentration of selenium in whole blood correlates well with the activity of GSH-Px, the percentage of selenium in erythrocytes that affects the GSH-Px varies widely and depends on the source of selenium [51]. Pavlata et al. [38] obtained similar results: positive correlation (r = 0.91) between selenium and GSH-Px in kids. According to [52], concentration of selenium in the blood of kids is about 40% lower than the concentration of selenium in their mother’s blood, which follows the results of the present research. These results should be considered when interpreting the concentration of selenium and GSH-Px in the whole blood of newborns.

### 4.3. Effects of Selenium on Blood Biochemical Indicators in Lambs

In lambs, the highest mortality and metabolic disorders occur during the first few days or weeks of life, mainly due to inadequate maternal feeding and poorly developed digestive systems of young lambs [53]. This problem, if not noticed and properly treated in time (late gestation; early lactation), can cause significant economic problems due to impaired productivity or mortality [54]. Analysis of biochemical and hematological indicators, i.e., metabolic profile together with antioxidant indicators, can help us in early detection of the mentioned problems and timely response in reducing or eliminating them. Selenium supplementation of ewes and lambs feed did not significantly influence mineral concentrations, but most of them were in the reference range [27]. The significantly higher (*p* < 0.05) concentration of albumin determined in the present study influenced the improved antioxidant activity of young lambs supplemented with selenium. According to [55], antioxidant activity is one of the most eminent functions of albumin. Decreased levels of albumin might act as a factor that contributes to the development of numerous oxidative stress-related disorders, such as diabetes and cancer. Similar findings to the present research have previously been determined by [37] on ruminants. Significantly increased concentration of globulin determined in the present study can improve innate and adaptive immune responses, and thus survival of lambs [56]. In numerous studies, it has been established that deficiency in selenium and vitamin E may compromise immune responses [57] and reduced immune function can increase the risk of infections. Furthermore, in the present study, significantly (*p* < 0.05) lower cholesterol concentration was found in lambs supplemented with inorganic selenium. Considering that selenium has a preventive effect on atherosclerosis, [58] initiated a series of studies in order to clarify the connection between antioxidants and blood cholesterol levels. Lower concentrations of triglycerides, cholesterol, and free fatty acids were found in the blood serum of rats fed cholesterol-rich rations with selenium supplementation [59]. In some studies [60], cholesterol concentration was not affected by selenium supplementation, and in others [61], cholesterol levels were higher with selenium supplementation. In agreement with the results of the present study, [62] found a significantly lower LDL concentration in lambs supplemented with selenium. In contrast, [63] found increased LDL concentration in lambs supplemented with selenium. Selenium supplementation in the present study resulted in a significant decrease in the activity of the enzymes AST and GGT. Liver enzymes, especially AST, ALT, and GGT, are commonly used to assess liver health or as indicators of liver damage and as a response to toxins [64]. Increased activity of the above enzymes is observed in animals treated with high doses of selenium over a longer period of time [46]. In the present study, the decreased activity of the enzymes AST and GGT in lambs supplemented with selenium was within the reference range [27] compared with increased activity of these enzymes in a control group of lambs without selenium supplementation. The presented activity of enzymes in lambs supplemented with selenium positively influenced their metabolism and had no toxic effect.

### 4.4. Effects of Selenium on Blood Hematological Indicators in Lambs

In the present study, a significant (*p* < 0.05) increase in WBC was observed in lambs supplemented with inorganic selenium. A higher percentage of lymphocytes and monocytes may lead to increased WBC counts in lambs supplemented with inorganic selenium. Similar results were reported by [65] in ewes, but without a significant increase. A significant increase in leukocyte count compared to the control group was observed when goats were orally administered a high dose of more than 2 mg Se/kg DM [63]. In many studies on different animals, the opposite effect of selenium supplementation on hematological indicators was found. Mohri et al. [66] found no significant effect on leukocyte counts in lambs supplemented with Se by injection. In the present study, significantly (*p* < 0.05) higher values of erythrocyte constant MVC, MCH, and MCHC were found in older lambs fed organic selenium supplements than the control group. Similar results were reported by [67] in goats supplemented with selenium (0.15 mg/kg BW) compared to goats fed a basal diet without Se supplementation. The erythrocyte constants MCV, MCH, and MCHC provide information about the average cell size, the hemoglobin content or the hemoglobin concentration in the erythrocytes. An increased MCV (macrocytosis) is an indicator of the cell’s regenerative response, while a decreased MCV (microcytosis) is often observed in some metabolic disorders, such as iron deficiency. An elevated MCH value may be an indicator of the presence of reticulocytosis (regenerative erythropoiesis) or hemolysis, while a decreased value may indicate an iron deficiency in the blood. According to [68], MCHC is considered the most accurate red cell indicator and may be elevated in hemolysis, while decreased values are observed in reticulocytosis and iron deficiency. Because of the close relationship between hemoglobin concentration and hematocrit, an elevated MCHC value is often an indicator of analytical error [69]. In the present study, a significant decrease (*p* < 0.01) in the number of platelets was observed in lambs supplemented with both organic and inorganic selenium supplements compared to the control group of lambs. A similar result was shown by [37] in lambs supplemented with organic selenium compared to the control group without selenium (705:822 × 10^9^/L). In the present study, a significantly (*p* < 0.05) higher number of eosinophils was observed in lambs with organic Se supplementation than in the control group without Se supplementation. These results are consistent with those of [66,70], who found significantly higher levels of eosinophils in lambs fed selenium and vitamin E without a clear mechanism of increase. In contrast, [71,72] found no difference in eosinophils between lambs and calves supplemented with Se compared to control ones.

### 4.5. Effectiveness of Selenium Transfer from Ewes to Lambs through Placenta and Milk

In newborns or during the suckling phase, the selenium supply of an organism depends on the availability of selenium in the body of the mothers, considering that selenium passes through the placental barrier, and that the transfer through colostrum and milk is possible. Transfer of selenium from mothers to calves through the placenta is more efficient than transfer through milk [73]. In ruminants, transfer of selenium through placenta occurs even when there is a shortage of this microelement in mothers, who, at the expense of their needs, provide selenium to fetuses [28]. In animals, and in humans, there is a decline in selenium concentrate in plasma as the gestation progresses, and as the fetus grows and increases its mass [74]. Selenium in colostrum and milk contributes to accelerated fetal growth, which is the reason for the increased selenium demand in late gestation [75]. These facts indicate the importance of the selenium addition before parturition [73] and after parturition [76] in order to maintain the concentration of selenium in the mothers and their progeny [9]. Hefnawy et al. [74] found higher (*p* < 0.01) concentrations of selenium in colostrum in a group of sheep fed with added selenium than in the control group. Also, they noted a good relationship between selenium concentration in plasma of sheep mothers and those in milk (r = 0.66 to 0.95; *p* < 0.05). Selenium concentration in milk was significantly higher (*p* < 0.01) in sheep that had the addition of selenium compared to those of the control group, which agrees with our research. As expected, the authors found that the relationship between the concentration of selenium in the milk of ewes and their lambs was positive in all groups (r = 0.57–0.73; *p* < 0.05), and these results are in accordance with the research of [77]. Similarly, as in the present research, no significant correlation (*p* > 0.05) between selenium concentration in plasma of mothers and their lambs was found, which indicates that young lambs depend on the selenium from mothers’ milk. Misurova et al. [52] found a significant positive correlation between the concentration of selenium in the blood of goats and their kids (r = 0.73; *p* < 0.01). The above authors determined a significantly high correlation (*p* < 0.01) between the concentration of selenium in the blood of goats and their kids using analyses of the regression line and the correlation coefficients. Stewart et al. [8] also found that a more effective transfer of selenium from whole blood of ewes to the whole blood of lambs and from ewes’ serum to serum of lambs occurs with the addition of selenium yeast to ewes’ food compared to sodium selenite. Although at least 15 selenium species have been documented, in selenized yeast the most important form is SeMet, and it is primarily responsible for the effective transfer of selenium [78]. Due to the biological similarities of selenomethionine and methionine, nonspecific replacement is possible. In other words, placenta does not differentiate between the two forms, and consequently SeMet is more effectively transferred to the fetus along with an amino acid transmitter, instead of independently. Therefore, there is a higher capacity of selenium accumulation in the blood of lambs of the ewes that had supplement selenium yeast in food compared to lambs whose mothers had an addition of inorganic selenium (sodium selenite), which corresponds to the results of this research.

### 4.6. The Relationship between Antioxidant Indicators in the Blood of Ewes and Their Lambs

The quality of selenium transfer from ewes to lambs and its impact on the antioxidant indicators are indicated by the high correlation coefficients, which were significant for GSH-Px and MDA concentrations in whole blood of group II lambs in the first sampling (r = 0.91, r = 0.98; Table 6 and Table 8). In accordance with these results, a good correlation between the activity of GSH-Px in the blood of goats and their kids was determined by [38]. In the mentioned research, a significant positive correlation (*p* < 0.01) (r = 0.77) between GSH-Px activities in the blood of goats and their kids was determined. Juniper et al. [13] recorded a linear effect (*p* = 0.102) of selenium yeast addition to lambs’ feed on TBARS value or MDA concentration in muscle. Comparing TBARS value and selenium concentration in longissimus thoracis, they found a decrease in TBARS with an increase in selenium level in the tissue (R2 = 0.302). The highest value of TBARS was recorded in the control group of lambs, without the addition of selenium in feed mixture, which suggests greater lipid oxidation.

## 5. Conclusions

The present study suggests that selenium dietary supplementation of dams in late gestation, and later of their lambs, improves oxidative stability, glutathione peroxidase, and superoxide dismutase activity, and reduces lipid peroxidation in ewes and lambs. The organic selenium source had a stronger effect on the increase of selenium concentration and glutathione peroxidase activity in the blood of ewes and lambs. Significant differences were found between studied antioxidant indicators in the blood of ewes and lambs considering selenium source (selenized yeast:sodium selenite) in their rations. In general, selenium addition (organic or inorganic) at a concentration of 0.3 mg/kg of dams’ and lambs’ feed mixture had a positive effect on the metabolism and immune function of the lambs without any negative effects. Given the positive response, and increased selenium concentration in the blood of younger and older lambs born to supplemented ewes, selenium is well transferred through the placenta and milk. Prepartum and postpartum selenium supplementations are important to maintain selenium concentrations during the demanding production phases of late gestation and early lactation in ewes and their lambs during the critical first weeks of life.

## Figures and Tables

**Table 1:** Ingredients and chemical composition of the basal feed mixture.

Component (%)	%
Corn	51.1
Wheat flour	15
Molasses	4
Yeast	3
Dehydrated alfalfa meal	2
Sunflower meal	9
Soybean meal	9
Animal salt	0.4
Limestone	2
Monocalcium phosphate	1
CMR	2.5
Premix	1
Basic Chemical Composition	%
Dry mater	88.0
Crude protein	17.7
Crude fiber	6.4
Crude fat	3.0
Ash	7.0
Metabolize energy (MJ/kg)	11.2

**Table 2:** Indicators of antioxidant status in whole blood of late-gestation ewes and lactating ewes.

Indicator	R.S.	Group			SEM	*p*-Value		
		I	II	III				
		Mean	Mean	Mean		Group	R.S.	R.S.×G.
Se, μg/L	L.G.	96.86 ^A^	157.56 ^B^	141.01 ^C^	5.71	<0.001	0.073	0.666
	L.	93.37 ^A^	143.18 ^Ba^	131.37 ^Bb^	4.79			
MDA, nmol/mL	L.G.	32.49 ^a^	20.13 ^b^	30.22 ^ab^	2.31	0.048	0.550	0.086
	L.	29.78	27.95	29.22	0.69			
GSH-PX, μkat/L	L.G.	581.18 ^A^	899.85 ^B^	771.67 ^B^	41.59	<0.001	0.063	0.008
	L.	569.08 ^A^	1054.91 ^B^	884.65 ^C^	57.55			
SOD, U/mL	L.G.	759.76	741.76	849.70	29.53	0.373	0.007	0.731
	L.	867.70	861.70	909.67	27.67			

Mean-mean value; SEM-mean standard error; I-control group; II-addition of organic selenium supplement; III-addition of inorganic selenium supplement; G.-group; R.S-reproductive status; L.G.-late gestation; L-lactation; ^a,b,^ *p* < 0.05; ^A,B,C,^ *p* < 0.01 differences between treatments; Se-selenium; MDA-malondialdehyde; GSH-Px-glutathione peroxidase; SOD-superoxide dismutase.

**Table 3:** Indicators of antioxidant status in whole blood of lambs at different ages.

Indicator	A. Days	Group			SEM	*p*-Value		
		I	II	III				
		Mean	Mean	Mean		Group	A.	G.×A.
Se, μg/L	23	48.21 ^Aa^	63.62 ^Ba^	56.76 ^b^	1.74	<0.001	<0.001	0.010
	63	58.62 ^A^	89.23 ^Ba^	80.71 ^Bb^	2.70			
MDA, nmol/mL	23	33.28	28.21	30.26	1.40	0.050	0.048	0.689
	63	28.95	25.31	22.67	3.14			
GSH-PX, μkat/L	23	590.65 ^A^	1023.78 ^Ba^	928.94 ^Bb^	52.01	<0.001	0.045	0.490
	63	596.80 ^A^	1017.51 ^Ba^	857.83 ^Bb^	36.47			
SOD, U/mL	23	950.15 ^Aa^	1183.13 ^B^	1115.97 ^b^	36.86	<0.001	<0.001	0.006
	63	1081.34 ^A^	1208 ^B^	1223.01 ^B^	20.90			

Mean-mean value; SEM-mean standard error; I-control group; II-addition of organic selenium supplement; III-addition inorganic selenium supplement; A.-age; G.-group; ^a,b,^ *p* < 0.05; ^A,B,^ *p* < 0.01 differences between treatments; Se-selenium; MDA-malondialdehyde; GSH-Px-glutathione peroxidase; SOD-superoxide dismutase.

**Table 4:** Blood biochemical indicators in lambs.

Indicator, mmol L^−1^	A. Days	Group			SEM	*p*-Value		
		I	II	III				
		Mean	Mean	Mean		Group	A.	G.×A.
Fe, µmol L^−1^	23	23.63	35.11	28.64	3.79	0.702	0.104	0.457
	63	35.92	36.16	39.57	2.31			
P	23	3.41	3.69	3.49	0.05	0.103	0.002	0.738
	63	3.14	3.32	3.28	0.06			
Ca	23	2.92	3.14	3.08	0.03	0.050	<0.01	0.632
	63	2.56	2.71	2.59	0.05			
Na	23 63	149.56 148.11	149.90 144.10	149.60 144.20	0.51 0.89	0.139	<0.01	0.233
K	23 63	5.70 4.84	5.68 4.74	5.30 4.56	0.09 0.06	0.054	<0.01	0.781
Cl	23 63	105.44 107.55	106.90 105.30	107.10 103.74	0.50 0.82	0.540	0.377	0.119
GUK	23	6.42	6.08	6.15	0.12		<0.01	0.102
	63	4.66	4.86	5.05	0.09	0.803		
UREA	23	7.43	7.78	7.36	0.29		0.420	0.034
	63	6.68	7.05	8.24	0.23	0.367		
TPROT, gL^−1^	23	56.17	54.41	52.97	0.67			
	63	53.14	52.75	53.76	0.45	0.262	0.163	0.239
ALB, gL^−1^	23	28.86 ^a^	30.18 ^b^	30.17 _b_	0.25	0.004	<0.01	0.746
	63	30.93	32.19	31.58	0.27			
GLOB, gL^−1^	23	22.80 ^a^	24.23 ^ab^	26.44 ^b^	0.59	0.032	<0.01	0.094
	63	22.21	20.56	22.17	0.39			
CHOL	23	2.69 ^a^	2.46 ^ab^	1.96 ^b^	0.13	0.036	<0.01	0.186
	63	1.76	1.65	1.46	0.06			
TGC	23	0.56	0.59	0.53	0.04	0.369	0.520	0.033
	63	0.73	0.49	0.53	0.05			
CREA, µmol L^−1^	23 63	59.56 59.38	55.20 55.80	60.60 59.60	1.39 1.81	0.312	0.909	0.939
HDL	23	1.48	1.29	1.18	0.05	0.095	<0.01	0.402
	63	0.92	0.92	0.79	0.03			
LDL	23	0.96 ^a^	0.90 ^ab^	0.54 ^b^	0.08	0.039	<0.01	0.068
	63	0.61 ^Bb^	0.40 ^A^	0.42 ^a^	0.03			
AST, UL^−1^	23	136.90	137.89	128.13	5.27	<0.01	0.072	0.083
	63	185.20 ^A^	148.38 ^B^	121.34 ^C^	5.56			
ALT, UL^−1^	23	27.93	31.94	29.90	1.96	0.940	0.378	0.566
	63	35.57	29.58	35.29	3.52			
GGT, UL^−1^	23 63	128.18 ^A^ 72.03	86.34 ^B^ 69.66	84.60 ^B^ 73.24	4.45 2.90	<0.01	<0.01	<0.01
CK, UL^−1^	23 63	176.20 210.21	160.44 172.08	166.88 162.90	6.20 9.11	0.071	0.224	0.079

Mean-arithmetic mean; SEM-standard error of the mean; I-control group; II-addition organic selenium supplement; III-addition of inorganic selenium supplement; A.-age; G.-group; ^a,b,^ *p* < 0.05; ^A,B,C,^ *p* < 0.01; Fe-iron; P-inorganic phosphorus; Ca-calcium; Na-sodium; K-potassium; Cl-chloride; GUK-glucose; TPROT-total proteins; ALB-albumin; GLOB-globulin; CHOL-cholesterol; TGC-triglyceride; CREA-creatinine; HDL-high-density lipoprotein; LDL-low-density lipoprotein; AST-aspartate aminotransferase; ALT-alanine aminotransferase; GGT-gamma-glutamyl transferase; CK-creatine kinase.

**Table 5:** Blood hematological indicators in lambs.

Indicator	A. Days	Group			SEM	*p*-Value		
		I	II	III				
		Mean	Mean	Mean		Group	A.	G.×A.
WBC (x 109/L)	23	8.04 ^a^	9.91 ^ab^	13.07 ^a^	0.99	<0.01	0.824	0.525
	63	8.78	-	11.54	0.75			
RBC (x 1012/L)	23	7.86	9.89	9.87	0.40	0.196	0.658	0.294
	63	8.44	9.63	8.06	0.49			
HGB, g/L	23	92.75	117.50	112.10	4.51	0.067	0.289	0.388
	63	107.20	124.60	106.35	4.53			
HCT, g/L	23 63	0.31 0.32	0.42 0.38	0.39 0.35	0.02 0.02	0.062	0.614	0.699
MCV, fL	23 63	39.23 ^a^ 38.12	41.94 ^b^ 38.69	39.46 ^a^ 37.68	0.42 0.26	0.004	0.002	0.435
MCH, pg	23 63	11.87 12.74 ^a^	11.91 13.71 ^A^	11.41 11.30 ^bB^	0.14 0.28	<0.01	<0.01	<0.01
MCHC, g/L	23	304.33	284.40	288.70	4.20		<0.01	0.036
	63	335.36 ^AB^	360.66 ^A^	229.90 ^B^	9.03	0.026		
PLT (x 109/L)	23	901.66	848.50	894.50	24.61		0.082	<0.01
	63	1275.20 ^A^	873.71 ^B^	689.24 ^C^	53.40	<0.01		
		Distribution of Leukocytes %						
Segmented neutrophil	23 63	50.62 36.50	43.27 44.50	45.92 38.33	2.69 1.29	0.856	0.019	0.122
Band neutrophil	23 63	0.13 0	00.27 0	0.16 0	0.07 0	0	0	0
Lymphocytes	23 63	48.75 62.75	53.54 53.45	52.16 65.00	2.71 1.75	0.419	0.007	0.128
Eosinophil	23 63	0.25 0.25 ^A^	2.09 2.00 ^B^	1.16 0.25 ^A^	0.38 0.19	0.016	0.627	0.259
Monocyte	23 63	0.12 0	0.09 0	0.16 0	0.08 0	0	0	0
Basophil	23 63	0.12 0	0.27 0.27	0.41 0.25	0.09 0.08	0.375	0.343	0.761

Mean-arithmetic mean; SEM-standard error of the mean; I-control group; II-addition organic selenium supplement; III-addition of inorganic selenium supplement; A.-age; G.-group; ^a,b,^ *p* < 0.05; ^A,B,C^ *p* < 0.01; WBC-white blood cells; RBC-red blood cells; HGB-hemoglobin; HCT-hematocrit; MCV-mean corpuscular volume; MCH-mean corpuscular hemoglobin; MCHC-mean corpuscular hemoglobin concentration; PLT-platelet count.

**Table 6:** Correlation coefficients and regression equations between the activities of GSH-Px in the blood of ewes and lambs.

Correlation	Group	Repro. Status	GSH-Px, μkat/L Lamb					
			Group, Days					
			I		II		III	
			23	63	23	63	23	63
GSH-Px, μkat/L ewe	I	L.G.	0.83					
		L.		0.75				
	II	L.G.			0.91 ^a^			
		L.				0.82		
	III	L.G.					0.82	
		L.						0.82
Regression Equation								
Age, Days								
Group	Repro. Status	23		63				
I	L.G.	GSH-Px = 178.98 + 0.70 × GSH-Px		-				
	L.	-		GSH-Px = −0.02 + 1.04 × GSH-Px				
II	L.G.	GSH-Px = 534.21 + 0.43 × GSH-Px		-				
	L.	-		GSH-Px = 360.04 + 0.56 × GSH-Px				
III	L.G.	GSH-Px = 561.19 + 0.59 × GSH-Px		-				
	L.	-		GSH-Px = 212.23 + 0.76 × GSH-Px				

I-control group; II-addition organic selenium supplement; III-addition inorganic selenium supplement; L.G.-late gestation; L.-lactation; ^a^ *p* < 0.05; GSH-Px-glutathione peroxidase.

**Table 7:** Correlation coefficients and regression equations between activity of SOD in the blood of ewes and lambs.

Correlation	Group	Repro. Status	SOD, μkat/L Lamb					
			Group, Days					
			I		II		III	
			23	63	23	63	23	63
SOD, μkat/L ewe	I	L.G.	0.64					
		L.		0.50				
	II	L.G.			0.62			
		L.				0.74		
	III	L.G.					0.42	
		L.						0.73
Regression Equation								
Age, Days								
Group	Repro. Status	23		63				
I	L.G.	SOD = 737.37 + 0.280 × SOD		-				
	L.	-		SOD = 923.22 + 0.182 × SOD				
II	L.G.	SOD = 687.71 + 0.613 × SOD		-				
	L.	-		SOD = 1063.6 + 0.150 × SOD				
III	L.G.	SOD = 687.52 + 0.469 × SOD		-				
	L.	-		SOD = 1047.5 + 0.202 × SOD				

I-control group; II-addition organic selenium supplement; III-addition of inorganic selenium supplement; L.G.-late gestation; L.-lactation; SOD-superoxide dismutase.

**Table 8:** Correlation coefficients and regression equations between the concentration of MDA in blood of ewes and lambs.

Correlation	Group	Repro. Status	MDA, nmol/mL Lamb					
			Group, Days					
			I		II		III	
			23	63	23	63	23	63
MDA, nmol/mL Ewe	I	L.G.	0.18					
		L.		0.47				
	II	L.G.			0.98 ^a^			
		L.				0.71		
	III	L.G.					0.48	
		L.						0.44
Regression Equation								
Age, Days								
Group	Repro. Status	23		63				
I	L.G.	MDA = 28.545 + 0.145 × MDA		-				
	L.	-		MDA = 17.618 + 0.380 × MDA				
II	L.G.	MDA = 17.378 + 0.639 × MDA		-				
	L.	-		MDA = −15.04 + 1.290 × MDA				
III	L.G.	MDA = 18.016 + 0.337 × MDA		-				
	L.	-		MDA = −17.05 + 1.515 × MDA				

I-control group; II–addition organic selenium supplement; III-addition of inorganic selenium supplement; L.G.-late gestation; L.-lactation; ^a^ *p* < 0.05; MDA-malondialdehyde.

## Data Availability

The data presented in this study are available on request from the corresponding author.
